# Scientific response to the 2021 eruption of Nyiragongo based on the implementation of a participatory monitoring system

**DOI:** 10.1038/s41598-022-11149-0

**Published:** 2022-05-06

**Authors:** G. Boudoire, S. Calabrese, A. Colacicco, P. Sordini, P. Habakaramo Macumu, V. Rafflin, S. Valade, T. Mweze, J.-C. Kazadi Mwepu, F. Safari Habari, T. Amani Kahamire, Y. Mumbere Mutima, J.-C. Ngaruye, A. Tuyishime, A. Tumaini Sadiki, G. Mavonga Tuluka, M. Mapendano Yalire, E.-D. Kets, F. Grassa, W. D’Alessandro, S. Caliro, F. Rufino, D. Tedesco

**Affiliations:** 1 Université Clermont Auvergne, UCA, CNRS, IRD, OPGC, Laboratoire Magmas et Volcans, 6 avenue Blaise Pascal, 63178 Aubière, France; 2 Istituto Nazionale di Geofisica e Vulcanologia - Sezione di Palermo, Palermo, Italy; 3 United Nations Development Programme, Goma, Democratic Republic of Congo; 4 Concern Worldwide, Goma, Democratic Republic of Congo; 5 Observatoire Volcanologique de Goma, Goma, Democratic Republic of Congo; 6 Departamento de Vulcanología, Instituto de Geofísica, Universidad Nacional Autónoma de México, Mexico City, Mexico; 7 Centre d’Expertise Géologique, Goma, Democratic Republic of Congo; 8 Université de Goma, Avenue de la Paix, Goma, Democratic Republic of Congo; 9 Rwanda Mines, Petroleum and Gas Board, KN 4 Ave, Kigali, Rwanda; 10 Mission de l’Organisation des Nations Unies pour la stabilisation en RD Congo, Goma, Democratic Republic of Congo; 11 Istituto Nazionale di Geofisica e Vulcanologia - Osservatorio Vesuviano, Napoli, Italy; 12 Present Address: DiSTeM, Università degli Studi di Palermo, via Archirafi 36, 90123 Palermo, Italy; 13 Present Address: DISTABIF, Università degli Studi della Campania Luigi Vanvitelli, via Vivaldi 43, 81100 Caserta, Italy

**Keywords:** Natural hazards, Volcanology

## Abstract

The development of a resilient society is a major challenge for growing human population faced with abundant natural hazards. During and after the May 22, 2021 eruption of Nyiragongo, the local population was surprised and scared by the subsequent seismicity and associated surface fracturing, coupled with the alert of a possible new eruptive vent opening in Goma (Democratic Republic of Congo) and/or Gisenyi (Rwanda). The creation of a toll-free phone number enabled the population to record fractures and gas/thermal anomalies affecting the area. Such work was fundamental in enabling scientists and authorities to assess the associated risks. Crucially, gas data showed that the degassing through fractures did not represent direct transfer of magmatic volatiles but was more likely of superficial origin. Surprisingly, this participatory work revealed that the first fractures appeared several weeks before the eruption and their opening was not detected by the monitoring system. This firmly underlines the need for scientists to anchor citizen science in monitoring strategies.

## Introduction

Ongoing global population growth raises severe issues about how we can live in a sustainable world and face natural disasters. Nowadays, almost one in eight people live within 100 km of an active volcano^1^. The recent paroxysmal eruptions at Stromboli (Italy) during the summer of 2019, at White Island (New Zealand) in December 2019, and at Nyiragongo (Democratic Republic of Congo) in May 2021, have highlighted the limits of current high-tech monitoring techniques in volcano crisis management. In this respect, we need to accept these limits, seek to constantly push them back, and succeed in setting an efficient communication between scientists, local authorities, stakeholders, (social)-media and exposed populations to favour information dissemination, preparedness, and resilience^2^.

As a consequence, the questions ‘When and where exactly?’, ‘For how long?’ and ‘What intensity and kind of event?’ may be not directly addressed even with the best current cutting-edge techniques, monitoring networks and worldwide expertise. These uncertainties become a strong motivation and focus for the whole scientific community. This is one of the main challenges in natural risk reduction and related communication during, sometimes non-predictable, disasters, as testified by the Global Risk Assessment Framework (GRAF) initiative of the United Nations Office for Disaster Risk Reduction (UNDRR) which itself was born from the Sendai Framework for Disaster Risk Reduction 2015–2030.

**Figure 1:**
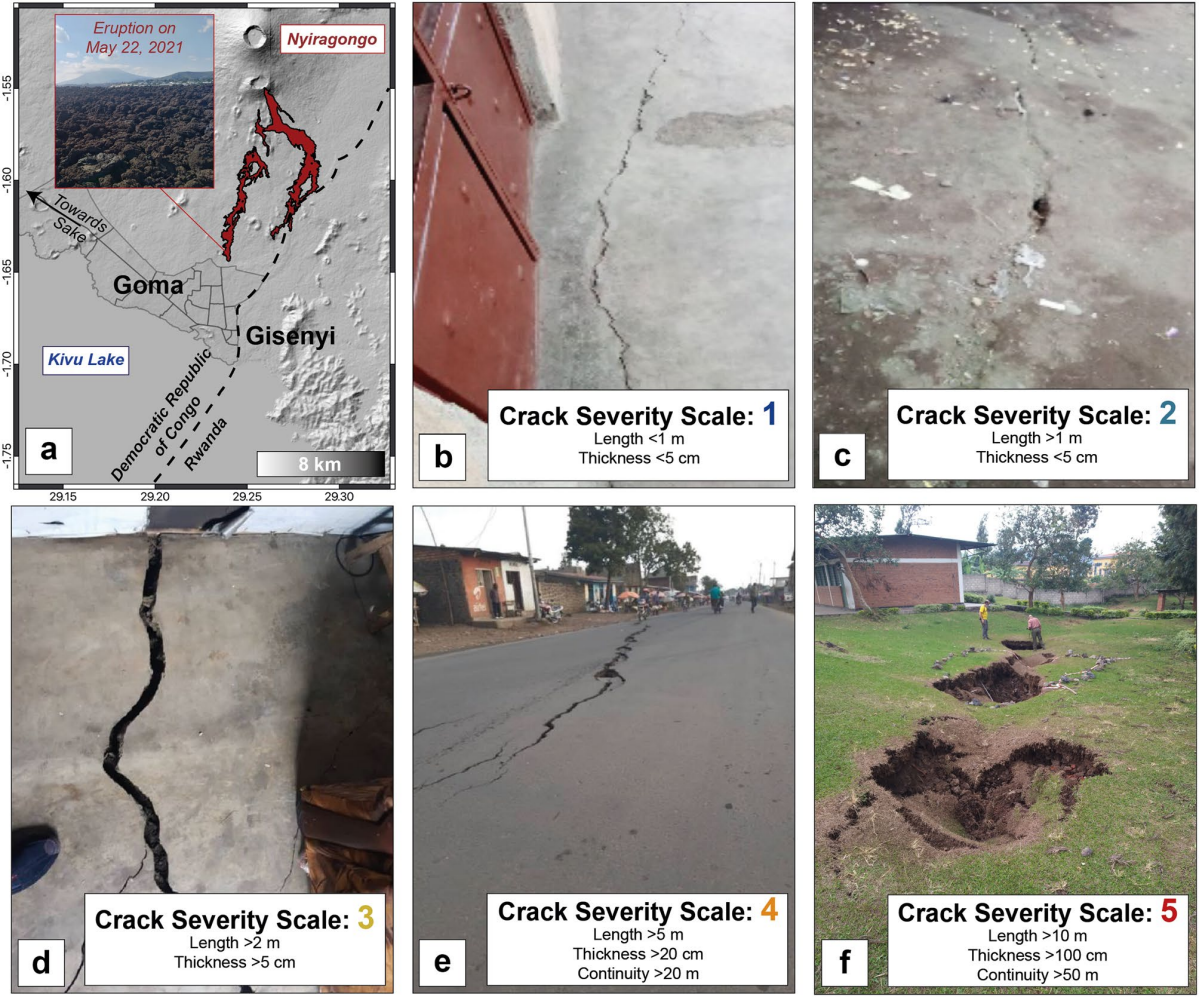
(**a**) Location map of the Nyiragongo volcano, the lava flows from the May 22, 2021 eruption (estimated volume not exceeding 10 Mm^3^ with respect to the minimum volume of 16 Mm^3^ estimated in the lava lake in 2020^9^; Burgi, pers. comm.), and the cities of Goma and Gisenyi. (**b**–**f**) Photos and severity index of the cracks across Goma and Gisenyi. Credits Attribution: Esri, USGS, ESA/CNES. Pictures from the authors.

The 2021 eruption of Nyiragongo (Democratic Republic of Congo: DRC) occurred on May 22 and lasted for just a few hours, but had a worldwide resonance^3^. Located in the North Kivu province and belonging to the East African Rift System (Fig. 1), the volcano is famous for its summit lava lake, the largest in the world, and is almost permanently active^4^.

The surroundings of the volcano are also in the sights of the international community for socio-political and humanitarian purposes. The area has been subject to chronic political and security instability due to the presence of numerous armed rebel groups and ethnic conflicts for more than 25 years. The North Kivu province is also the place of many epidemics such as cholera, measles and Ebola^5^. In this context, the area is characterized by a population explosion and urban development, especially in the neighbouring cities of Goma and Sake (DRC) as well as Gisenyi (Rwanda) (Fig. 1). This makes Nyiragongo one of the most dangerous and deadly in the world, with an extreme level of risk being amplified by the continuing seismic risk in the area due to rift-related tectonic earthquakes^4,6,7^.

The previous eruption on January 17, 2002 left 120,000 inhabitants homeless and killed at least 170 people^7^. An unusually long period of earthquakes and volcanic tremor, the appearance of fumaroles, the increase of soil temperature, and opening of cracks and/or smells of gas were reported in local testimonies in the year preceding the eruption^6^.

In this study, we show the benefits of the implementation of a participatory tracking and monitoring system of the fractures opening at Goma and Gisenyi caused by the seismic activity in the days following the 2021 eruption of Nyiragongo. This “citizen science” approach^8^ allowed (i) the rapid production of a variety of maps referencing the network of fractures in the urban areas, and (ii) the collection of local testimonies reporting the opening of some fractures in the weeks preceding the eruption notwithstanding the apparent absence of short-term precursors detected on monitoring signals.

Moreover, merging these results with the geochemical survey performed by a team of international and national scientists immediately after the production of these maps provided precious scientific results to identify (1) whether, within Goma and Gisenyi, gases escaping from the cracks could be at immediate risk for inhabitants, and (2) whether these gaseous emissions originated from the propagation and upwelling of a magma batch from depth (a dyke) beneath the area with the risk of a possible new eruptive vent opening directly within the cities or in their outskirts.

### Phenomenology of the 2021 seismovolcanic crisis

After the January 17, 2002 eruption, the eruptive activity at Nyiragongo was confined within the summit crater for 19 years with a permanent lava lake^4^. This reached a high and critical level prior to the 2021 eruption that led the scientific community to issue a call for vigilance regarding the current and near future activity of the volcano^9,10^. However, the opening of eruptive fissures on the volcano flanks on May 22, 2021 (Fig. 1) took by surprise the scientific community due to the apparent lack of short-term precursors preceding the eruption^3^. In a few hours, the lava reached the northern districts of Goma, destroying and or damaging more than 1000 households. Panic movements were reported in Goma causing, before the lava stopped a few hundred meters from the international airport, migration of people fleeing across the Rwanda border as well as the disappearance of many children who became separated from their families^11^.

Earthquakes were recorded beneath the Nyiragongo–Goma–Gisenyi–Lake Kivu area in the days following the eruption, mirroring what was observed after the 2002 eruption^6^. These earthquakes were strongly felt by the local population, generating panic and damaging local infrastructure and houses (Fig. 1). On May 27, 2021, due to the fear of a new eruptive vent possibly opening in Goma’s urban area resulting from the propagation of a dyke^12^, local authorities decided to transfer the population from a large part of Goma to the city of Sake (Fig. 1). More than 300,000 people were displaced for two weeks in unsatisfactory conditions (lack of infrastructure, shelter, cholera epidemic), during which time they awaited further instructions concerning the risk in Goma.

On May 24, 2021 (i.e., 2 days after the eruption), a participatory tracking and monitoring system of the fractures caused by the seismic activity was put in place by MONUSCO (Mission de l’Organisation des Nations Unies en République Démocratique du Congo) and PNUD (Programme des Nations Unies pour le Développement) with the support of the non-governmental organisation Concern Worldwide (see “Methods” for a complete description of the protocol; Supplementary Fig. S1), the Observatoire Volcanologique de Goma (OVG), and several local institutions. Goals were to build a database of fractures and gas/thermal anomalies within the urban areas of Goma and Gisenyi, and allow a team of national and international scientists to carry out gas and temperature measurements at these fractures. The area is well-known for CO_2_-rich gas pockets, locally called “mazuku”, which are considered one of the most persistent risks for the inhabitants of the area. CO_2_ is a colourless and odourless asphyxiant gas, denser than air, and which at concentrations higher than 10% will lead to coma and death in few minutes^13^. It was therefore essential to identify if, within Goma and Gisenyi, CO_2_ was escaping from the cracks.

### Insights from the participatory tracking and monitoring system of the fractures

**Figure 2:**
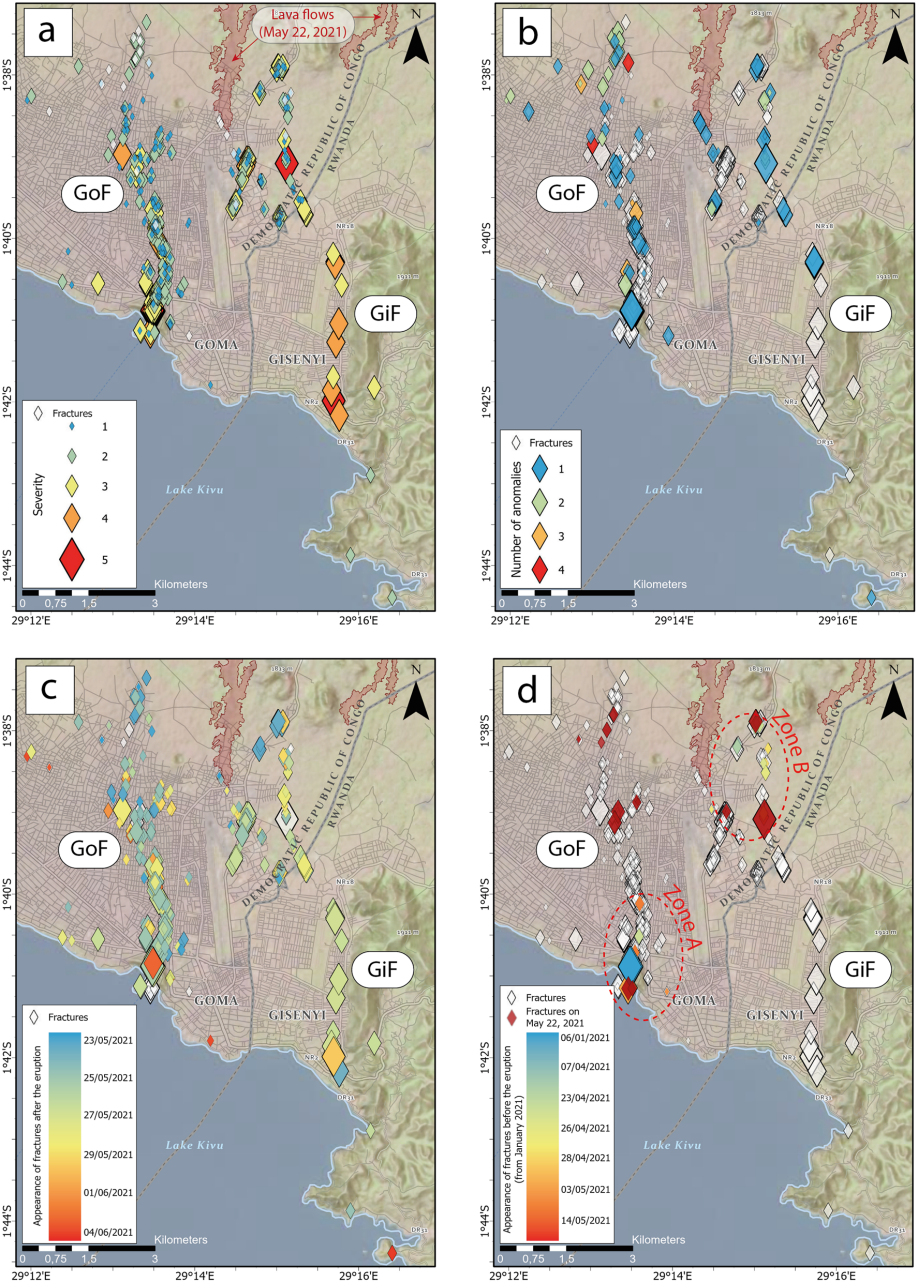
Maps produced from the results of the participatory system. (**a**) Location of the fractures classified by severity index. (**b**) Number of anomalies identified on fractures by the population of Goma and Gisenyi (uncoloured diamonds denote fractures without anomaly). Chronology of the appearance of fractures (**c**) after and (**d**) before the May 22, 2021 eruption. Credits Attribution: Esri, NASA, NGA, USGS; RMLUA, OpenStreetMap, HERE, Garmin, METI/NASA.

A total of 458 fractures (Supplementary Data S1) were identified by the population involved in the participatory tracking and monitoring system of the fractures. The map revealed two main parallel networks of fractures from North to South (Fig. 2). These fractures networks extended parallel to the rift axis^14^ and were reminiscent of the evolution of segmented continental rift basins^15^. The first network of fractures crossed the city of Goma (“GoF” stands for “Goma main network of Fractures”) with a severity index rarely exceeding 3 out of 5 (Fig. 2a) on the scale defined by the local representants and collaborators of the United Nations (see “Methods”). Several thermal and gas anomalies were reported by the population on the northern part of the “GoF” (Fig. 2b). The second network of fractures extended from the northern districts of Goma (namely “Munigi”) to Gisenyi (“GiF” stands for “Gisenyi main network of Fractures”) and was marked by a higher index of severity (> 4). Together with the severity index, the main difference between the “GoF” and the “GiF” was the absence of temperature anomalies reported by the population along the “GiF” (Supplementary Fig. S2).

In terms of the temporal evolution of fracture appearance, it is noteworthy that most of the “GoF” developed in the 72-h following the eruption (May 23–25), whereas most of the “GiF” developed later (May 24–29) (Fig. 2). More surprising were the reports of the appearance of a few fractures starting since April 23, 2021: 16 fractures that appeared between April 23 and May 3, 2021 were reported by the population at the time of the participatory tracking and monitoring system of the fractures. These fractures are concentrated in two distinct zones along both the “GoF” and the “GiF” (Zones A and B on Fig. 2). These brittle deformation events one-month prior to Nyiragongo’s eruption support the idea of a complex interplay between local processes and regional stress fields in controlling seismic and eruptive activity^4,6,9,16^. Interestingly, the OVG staff reported^17^ an unusual occurrence of hybrid seismic events^18^ around Nyamulagira and Nyiragongo between April 10 and May 6, 2021, peaking in a seismic swarm from April 21 to 25. This was the only relevant activity known in the months preceding the eruption.

### Gas and thermal measurements made on the networks of fractures

**Figure 3:**
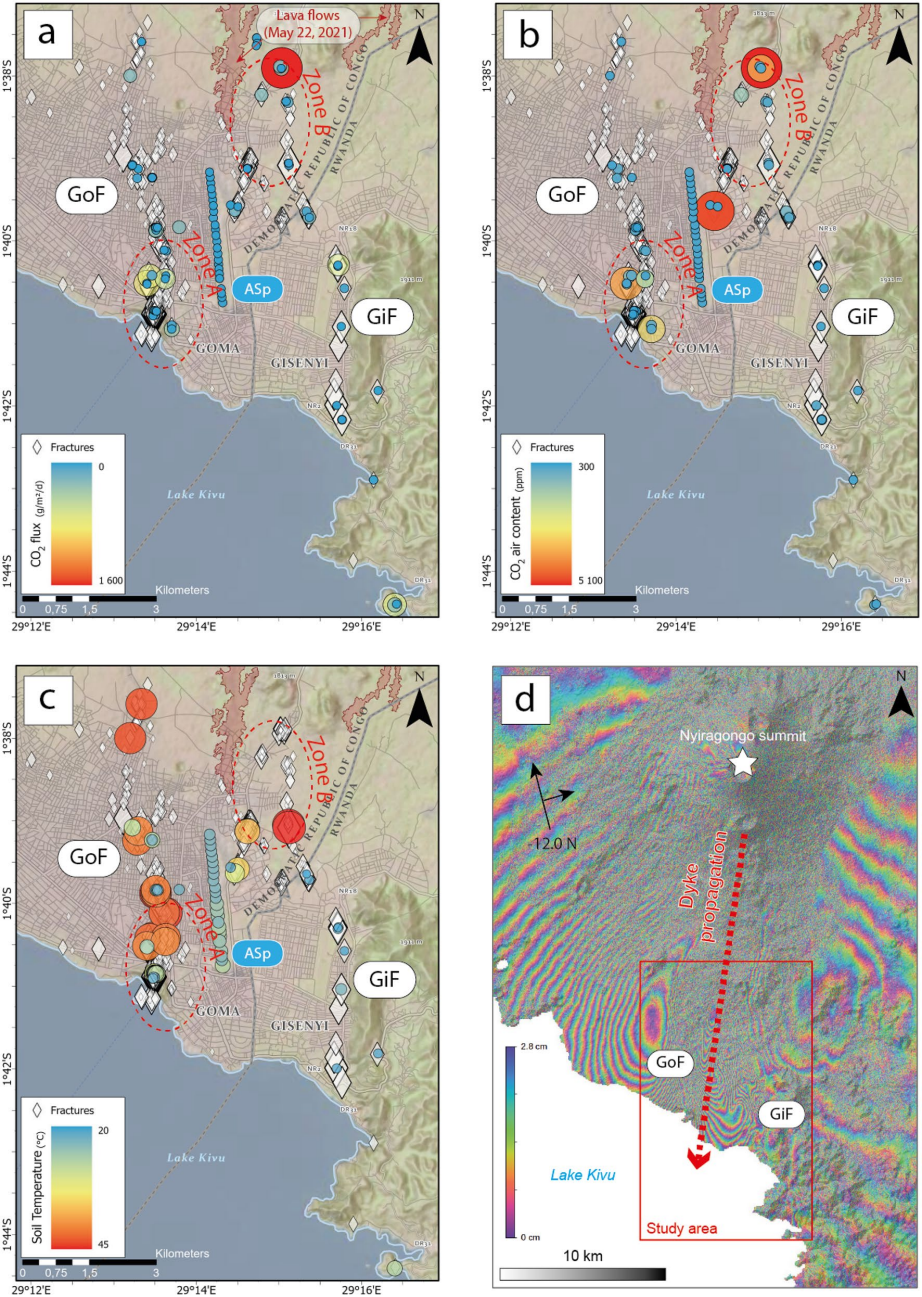
Maps produced from the results of the survey performed by the international scientific team and local institutes: (**a**) CO_2_ flux from fractures and soils, (**b**) CO_2_ content in the air close to the soil, (**c**) temperature within the fractures and in the soil (20 cm-depth). (**d**) Interferogram showing the ground deformation related to the dyke propagation. The large-scale deformation pattern (Supplementary Fig. S4) displays two lobes with opposite axis^34^. The propagation path is therefore interpreted to be nearly North–South, similar to the network of fractures. Credits Attribution: Esri, NASA, NGA, USGS; RMLUA, OpenStreetMap, HERE, Garmin, METI/NASA, ESA.

During June 8–19, 2021, with the help of the local population, we carried out temperature and gas measurements on accessible fractures (see “Methods”; Supplementary Data S2). First, a 100-m-resolution profile of soil CO_2_ flux, CO_2_ content in the air and soil temperature was constructed at Goma airport (“ASp” in the following standing for “Airport Soil profile”; Fig. 3). This was done to provide local background values (from the longest continuously vegetated area of Goma). Soil CO_2_ flux here ranged from 1.5 to 18.4 g m^2^ day^−1^, which is in the range of CO_2_ production from a wide variety of ecosystems (i.e., < 21 g m^2^ day^−1^)^19^. CO_2_ content in the air sample (< 400 ppm) did not exceed classical atmospheric values and soil temperature ranged from 22.5 to 26.9 °C. Similar temperature values were measured in fractures belonging to the “GiF” (23.8 ± 4.0(1σ) °C) (Fig. 3). Conversely, higher temperatures characterized the “GoF” (33.5 ± 3.3(1σ) °C) consistent with local reports of temperature anomalies along that system (Supplementary Fig. S2) and the potential path of a dyke at depth (Fig. 3).

Soil CO_2_ fluxes from fractures ranged from below the instrumental detection limit to 1572 g m^2^ day^−1^. High fluxes were mostly restricted to Zone A (175 ± 143(1σ) g m^2^ day^−1^) and B (252 ± 297(1σ) g m^2^ day^−1^), i.e., where the first fractures propagated before the eruption (Fig. 3). These two zones are (consequently) the main areas of CO_2_ anomalies in the air, with values up to 3900 and 5100 ppm, respectively. These values, which are close to the maximum recommended occupational exposure limit of 5000 ppm (as an 8-h total weight average in most jurisdictions), were not an immediate concern for the local population, but still deserved to be monitored in the next months. The telephone numbers of the OVG staff present on site were shared with home owners to allow the population to inform the observatory should there be any development or concern. In parallel with the analysis performed in the streets and homes of Goma, preventive actions related to the risk posed by CO_2_ emanations were disseminated to the population by the OVG staff: (i) ventilate the rooms as much as possible, (ii) elevate and move the beds away from the fractures, (iii) fill the cracks with sand and then cement and, (iv) get out quickly and contact the observatory in case of headaches.

**Figure 4:**
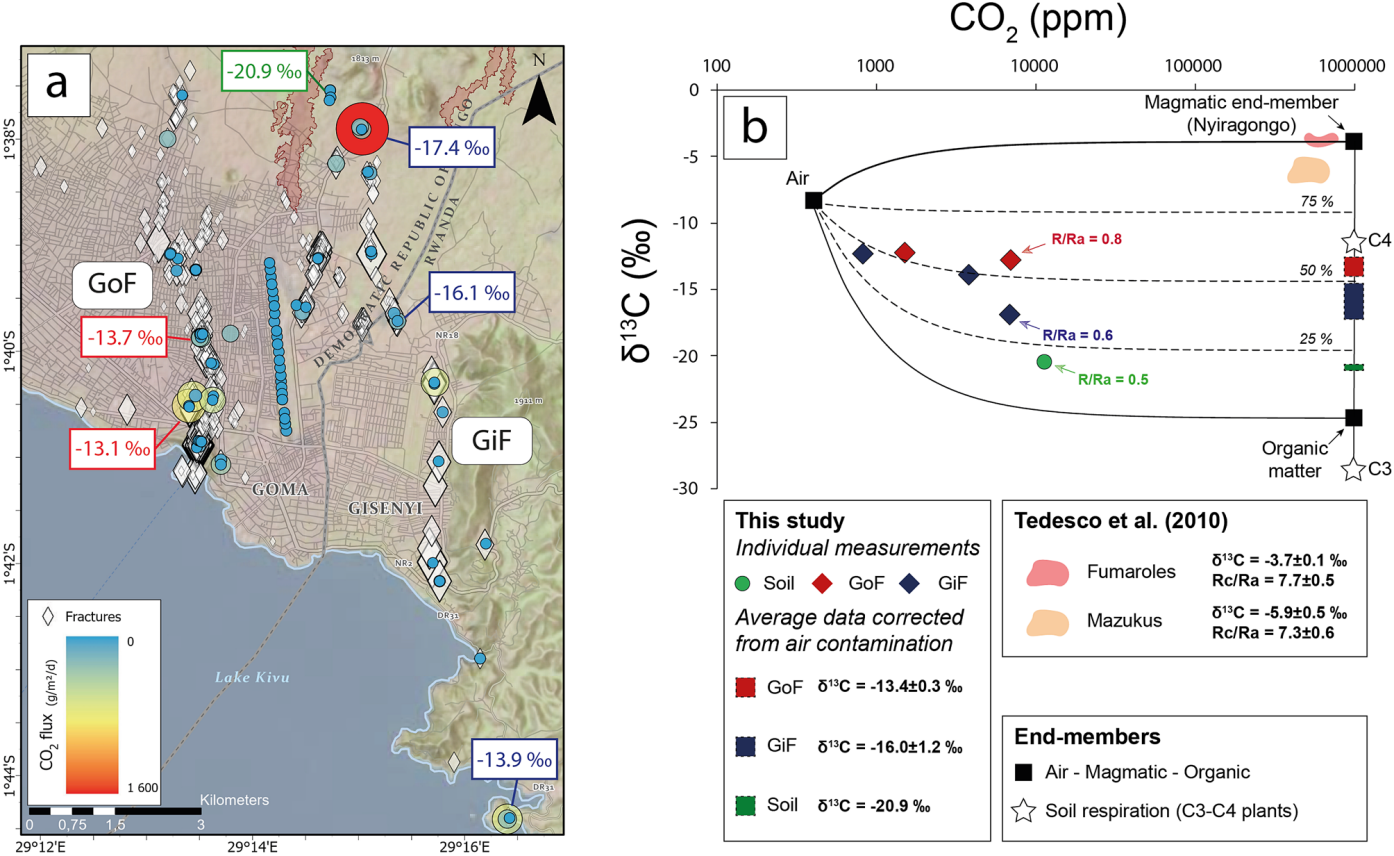
(**a**) Map of CO_2_ flux with carbon isotopes values. (**b**) CO_2_ content versus δ^13^C of CO_2_ from fractures. The end-members used in the mixing model are defined in the “Methods”. Credits Attribution: Esri, NASA, NGA, USGS; RMLUA, OpenStreetMap, HERE, Garmin, METI/NASA.

The δ^13^C values of CO_2_ sampled from the fractures, and corrected for air contamination (see “Methods”), had an average of − 13.4 ± 0.3(1σ)‰ within the “GoF” and − 16.0 ± 1.2(1σ)‰ within the “GiF” (Fig. 4a). The δ^13^C values became more negative with distance from Lake Kivu’s shores. These values are lower than previous measurements performed around Goma (values range from − 3.5 to − 6.8‰ in “mazuku” and fumaroles)^19^. Gases escaping from fractures did not show a magmatic signature (values range from − 3.5 to − 4.0‰ in fumaroles at Nyiragongo; Fig. 4b)^20^. We can reasonably discard the effect of soil respiration (C3–C4 plants)^21^ in such negative values knowing that (i) measurements were realized mostly in an urban setting without soil and vegetation (see pictures on Fig. 1), and (ii) gas samples were collected only on sites of high soil CO_2_ flux (> 54 g m^2^ day^−1^, i.e., more than twice the maximum value withheld for the CO_2_ production from a wide variety of ecosystems)^19^. Only one gas sample was collected in an unfractured area (in the north of Goma; Fig. 4a) characterized by the presence of vegetation and by a lower soil CO_2_ flux (32 g m^2^ day^−1^; cf. “Soil” on Fig. 4b) precisely to document the partial effect of soil respiration on δ^13^C values (δ^13^C < 20.9‰ in our case). ^3^He/^4^He values (R/Ra = 0.5–0.8) reveal a predominant crustal contribution (R/Ra = 0.05) in gas samples that are neither compatible with a potential mixing (about 50/50 based on δ^13^C values, only; Fig. 4b) between a gas marked by a magmatic–mantellic origin as measured in the surroundings of Goma (δ^13^C > − 6.8‰; R/Ra > 4.2)^20^ and crustal gases (δ^13^C < − 25‰; R/Ra < 0.05)^22^. These results strongly suggest that the CO_2_ emissions along these fractures were not directly released by magma in the dyke that supposedly propagated beneath the area (Fig. 3d, Supplementary Fig. S3) or to mantle outgassing along deep lithospheric structures. Instead, they support the idea that the opening of the fractures promoted the discharge of gas that has already acquired a negative carbon isotopic signature (δ^13^C from − 13.1 to − 17.4‰)^23^. This inherent feature may result from (i) the baking of a mixture of underlying sediments-carbonates^24,25^ or (ii) a Rayleigh fractional condensation process of the CO_2_ already stored in the uppermost part of the crust favoured by groundwaters and intense tropical rainfalls^26,27^.

### Lessons learned and potential future applications

The ability of the population to participate in mapping fractures and anomalies within the urban areas of Goma and Gisenyi provided crucial prerequisites to the work carried out by the scientific teams responding to the post-eruptive event crisis. This participatory system allowed the team deployed on site to work efficiently. It would have been time-consuming (and thus counter-productive due to the emergency situation) and less accurate (due to the difficulties of accessing certain locations safely) to perform this mapping of the fractures by ourselves. Moreover, this “citizen science” approach allowed us to develop a connection with the local populations that was greatly appreciated by both parties as well as necessary for the development of a trust relationship between scientists and populations. Many of the people in whose houses the measurements were performed, or to whom the preventive actions were communicated, expressed their satisfaction with the presence of the observatory’s staff in the field.

The strategy for geochemical measurements was then based on these maps derived from citizen participation and permitting a better response to the 2021 volcano-tectonic event. Isotopic data of gaseous emissions released by fractures in the two urban areas were consistent with increased crustal degassing through the newly opened fractures rather than new magma degassing. This finding minimizes, for this specific case study, the probability of occurrence of a new eruptive event within the urban areas as a consequence of dyke intrusion. Content of CO_2_ in the air inside houses targeted during the geochemical survey rarely exceeded 1000 ppm (less than 10% of the dataset indicates CO_2_ content above 1000 ppm with a maximum value of 5100 ppm, slightly above the maximum recommended occupational exposure limit). Even though these values were not of immediate concern for the population, preventive actions were nevertheless disseminated locally considering that CO_2_ concentrations were likely to increase during the night, as observed in local “mazuku”^13^.

The recognition by the population that fractures were forming and extending days to weeks prior to the eruption is one of the crucial points that emerged from this study, but needs further research and verification prior to any future eruptive episodes. Such work can be assured by the development of a closer relationship between authorities, scientists, and the local population. With respect to the apparent absence of short-term precursors recorded by the monitoring network, our findings highlight the exigency to sustainably anchor citizen science in future monitoring strategies^27^, especially in an area where (i) the deployment of a multidisciplinary operational monitoring network is challenging, and (ii) the participation of the population may actively contribute to warning systems that can mitigate the impacts of natural disasters, and improving its resilience in the face of future ones. More generally, in provinces at risk of natural disasters, our study strongly encourages the development of initiatives (communication operations, participatory systems) that aim at strengthening relationships between citizens, scientists, and authorities. These initiatives are even more crucial during emergency periods when the lack of communication may exacerbate counterproductive tensions between the different parties. This was evident after the 2021 eruption of Nyiragongo^3^ but is also currently the case at Vulcano and Stromboli (Italy) since the implementation of access restrictions due to volcanic unrest and unsafe conditions can be misunderstood by the local population^28^.

The scientific survey performed at Goma and Gisenyi have emphasised the need to develop a well-suited operational geochemical monitoring network there with two main objectives. The first one is to forecast potential variations of soil degassing linked to the regional volcano-tectonic activity (the highest soil CO_2_ flux has been measured in the zones where fractures started opening weeks before the eruption; Fig. 3a). Such an improvement of the monitoring network should also focus on the control of air quality in houses affected by the opening of the fractures or built-in areas of intense soil degassing. In both cases, such a development again requires a strong synergy and buy-in between scientists, authorities and local population.

## Methods

### Participatory mapping

The participatory tracking system of the fractures caused by the seismic activity was put in place by MONUSCO (Mission de l’Organisation des Nations Unies en République Démocratique du Congo) and PNUD (Programme des Nations Unies pour le Développement) with the support of the non-governmental organization Concern Worldwide.

The key point of this participatory system was the setting up of a toll-free number (with a specially purchased SIM card) allowing the inhabitants of Goma and Gisenyi to report the presence of fractures by transmitting via WhatsApp (i) the GPS coordinates of the fractures, (ii) pictures to establish the degree of severity (Fig. 1), (iii) date of occurrence, and (iv) related observed anomalies (unusual smell, gas emissions, smoke emissions, anomalous temperature). A flyer describing the procedure and written in English, French, and Swahili (Supplementary Fig. S1) was disseminated to the citizens by both personal acquaintances and key actors, such as local businesses, the Stabilization Support Unit (SSU) of the MONUSCO, and religious organizations which are locally very much listened to and respected. Then, the flyer spread by word of mouth. The data were then continuously collected by a team of 6 people from UNDP including the manager A. Colacicco, each taking turns every 4 h.

On May 29, 2021, the first map of the fractures was produced and communicated to the volcanologists. The toll-free number triggered a constant flow of information which allowed daily updates of the database and of the map in the following days. On June 1, 2021, the UNDP and the SSU managed to put together and train a team of 44 volunteers to carry out a mobile-based data collection at Goma (June 3–5, 2021) in order to cross-check the information received via WhatsApp, complete the mapping of the whole city, and define a severity index of the fractures. This index (from 1 to 5) was defined on the basis of the length, thickness and continuity of the fractures (i.e., spatial interconnectivity between them). In parallel, geologists from the RMB were deployed to Gisenyi to map the fractures. The 458 fractures presented in this study were identified by the population involved in the participatory tracking and monitoring system and cross-checked by the team of volunteers. All “questionable” data, such as the appearance of fractures before the eruption, were double-checked by the volunteer in charge of the zone. This volunteer was instructed to verify the date of appearance of the fractures in presence of a minimum of 2 witnesses.

No data about the human participants were collected for the following three reasons. (1) The study on fractures is based on observation of hard facts, therefore those should not be influenced by the social status of the observer. (2) A participatory system should be accessible, quick an easy. We cannot take too much time from citizens; therefore, only straight forward information, which are strictly related to the focus of the research, have to be asked. (3) The survey was carried out in a time of emergency while the cities of Goma and Sake were shaken by continuous earthquakes. In order to guarantee the safety of the people who are sharing data while standing right next to potentially dangerous spots it is crucial to minimize their time in the zone of danger as much as possible. Therefore, the survey needed to be straight to the point.

### Field measurements

Soil CO_2_ flux measurements were performed following the Accumulation Chamber Method^29^ by the way of a West System device composed by an accumulation chamber (type A) connected to a temperature-stabilized LI-COR LI-830 infrared sensor (0–20,000 ppm CO_2_) compensated in pressure and temperature. The sensor has a detection limit of 1.5 ppm on CO_2_ content and a 3% accuracy of reading. The flux (g m^−2^ day^−1^) of each measurement was elaborated from the related regression line (ppm s^−1^) by using the FluxRevision free software, knowing the air temperature, barometric pressure and volume of the accumulation chamber. Barometric pressure and air temperature were recorded for each point of measurement by the mean of a Kestrel 5500 Weather Meter with an accuracy of 1.5 mbar and 0.5 °C, respectively.

Atmospheric CO_2_ measurements were performed at the soil-air interface above the fractures with a low power NDIR (Non-Dispersive InfraRed) diffusive portable gas sensor iGas CO_2_ (0–5%) with an accuracy of 100 ppm on CO_2_. The temperature within the fractures was taken between 10 and 20 cm-depth according to the ability to insert the probe inside. Type K thermocouple was used with an accuracy of 2.2 °C on temperature.

Gas escaping from fracture was sampled essentially on sites of high flux to increase the amount of available CO_2_ for laboratory analysis. The gas was collected directly at the output of the accumulation chamber by the mean of a three-ways glass system connected to 12 mL Exetainer glass vials and in two-stopcock glass bottles. Vials and bottles were pre-cleaned injecting 500 mL of the sample gas in order to remove the initial volume of air trapped in the vials.

### Laboratory analysis

CO_2_ content in glass vials was analyzed at the INGV Palermo (Italy) by the use of a micro module gas chromatographer (MicroGC 3000) equipped with a Poraplot U column (15 m) fluxed by Ar. Analytical precision was better than 3% of the measurement. The detection limit was about 50 ppm.

Analysis of carbon isotopes were performed at the INGV Palermo (Italy) with a Thermo Delta Plus XP CF-IRMS with a precision of 0.15‰ on δ^13^C and coupled with a Thermo TRACE Gas Chromatograph (GC) and a Thermo GC/C III interface. The analysis of the carbon isotopes of CO_2_ were replicated (reproducibility better than 0.7‰ on δ^13^C for CO_2_ content greater than 1500 ppm) at the Laboratory of Fluid Geochemistry of the INGV Osservatorio Vesuviano, using a Thermo Delta Plus XP continuous flow mass spectrometer coupled to the GasBench II device with a reproducibility better than 0.20‰ on δ^13^C.

Abundance and isotopes compositions of He were determined by the mean of a Helix SFT-GVI split flight tube mass spectrometer at the INGV Palermo. The analytical error on He isotopes analyses was less than 0.4%. The purification procedure for helium and the full procedure is the same as described in Boudoire et al.^30^.

### Data elaboration

The ^3^He/^4^He (R) is expressed as R/Ra (Ra being the He isotopes ratio of air equal to 1.39 × 10^–6^). δ^13^C of CO_2_ refers to the ratio of stable isotopes ^13^C/^12^C (reported in part per thousands) and normalized to Pee Dee Belemnite (PDB) reference standard.

Mixing curves on Fig. 4b were defined by using (i) an averaged δ^13^C value of − 3.7‰ from Nyiragongo fumaroles (at 100% CO_2_) for the magmatic end-member^20^, (ii) a biogenic δ^13^C value of − 24.7‰ from charcoal (at 100% CO_2_) for the biogenic end-member^31^, and (iii) an atmospheric value (at 398 ppm CO_2_ measured by gas chromatography from a glass vial collected at Goma) of − 8.4‰ in accordance with recent measurements performed in the atmosphere^32^.

Individual measurements of δ^13^C of CO_2_ were corrected from air contamination (aerated fractures) by looking for the intercept of the linear regression between 1/CO_2_ and δ^13^C of the sampled gas and the air. The intercept reflects the δ^13^C value at 100% of CO_2_ (i.e., without air contamination).

Maps were created using ArcGIS^®^ software (ArcGIS Pro 2.8) by Esri in the Projected and Geographic Coordinate System WGS 1984 Web Mercator Auxiliary Sphere and WGS 1984 UTM Zone 35N (Landsat). ArcGIS^®^ and ArcMap™ are the intellectual property of Esri and are used herein under license. Copyright © Esri. All rights reserved. For more information about Esri^®^ software, please visit www.esri.com.

Interferograms were generated from Sentinel-1 (ESA Copernicus) products to visualize the large-scale deformation related to the dike intrusion. Sentinel products were freely downloaded from the Copernicus Open Access Hub (https://scihub.copernicus.eu/), and processed by the volcano monitoring system MOUNTS^33^. The interferograms are computed by DInSAR (Differential Synthetic Aperture Radar Interferometry), from two products acquired on 2021-04-21 16:20 UTC and 2021-05-01 16:20 UTC, and two other products acquired on 2021-05-19 16:20 UTC and 2021-05-31 16:20 UTC (ascending orbit, relative orbit number 174). Interferograms are displayed with a wrapped interferometric phase, whereby each fringe (full phase cycle ranging between 0 and 2π) corresponds to 2.8 cm in the radar line-of-sight.

## Supplementary Information

Supplementary Figures.

Supplementary Table 1.

Supplementary Table 2.

## Data Availability

All data are available in the main text or in the Supplementary Material 1, and Supplementary Tables 1 and 2. In Figs. 1a, 2, 3 and 4a the Digital Elevation Model was processed from the STRM data of the USGS EROS Data Center and the shapefile for lava flows comes from UNITAR-UNOSAT products based on a Sentinel-1 image acquired on May 25, 2021. The background maps on Figs. 2, 3 and 4a are extracted from the ESRI World Terrain Base (ArcGIS) and the Landsat 8 Operational Lan Imager (OLI) and Thermal Infrared data of the USGS EROS Data Center (LC08_L1TP_173061_20200817_20200822_01_T1). In Fig. 1a–f, pictures are from A. Colacicco, P. Sordini, and G. Boudoire. In Fig. 3d, the interferogram is generated from Sentinel-1 (ESA Copernicus) products. Sentinel products were freely download from the Copernicus Open Access Hub (https://scihub.copernicus.eu/).
