# Supplementary material for: Scientific response to the 2021 eruption of Nyiragongo based on the implementation of a participatory monitoring system
Source: Sci Rep. 2022 May 6;12:7488. doi: 10.1038/s41598-022-11149-0 (PMC9076609; doi:10.1038/s41598-022-11149-0)
Supplement: Supplementary file 1 — Supplementary Figures. [file 41598_2022_11149_MOESM1_ESM.docx]

Supplementary Material 1 for

Scientific response to the 2021 eruption of Nyiragongo based on the implementation of a participatory monitoring system

G. Boudoire, S. Calabrese, A. Colacicco, P. Sordini, P. Habakaramo, V. Rafflin, S. Valade, T. Mweze, J.-C. Kazadi, F. Safari, T. Amani, Y. Mutima, J.-C. Ngaruye, A. Tuyishime, F. Grassa, A. Sadiki, G. Mavonga, M. Yalire, E.-D. Kets, W. D’Alessandro, S. Caliro, F. Rufino, D. Tedesco

Correspondence to: [guillaume.boudoire@uca.fr](mailto:guillaume.boudoire@uca.fr), [sergio.calabrese@unipa.it](mailto:sergio.calabrese@unipa.it) and [dario.tedesco@unicampania.it](mailto:dario.tedesco@unicampania.it)

**This PDF file includes:**

Figs. S1 to S3

Captions for Data S1 to S2 (Supplementary Tables 1 and 2)

Fig. S1. Flyer describing the procedure of the participatory system that was disseminated to the citizens by local businesses, religious organizations and the Stabilization Support Unit (SSU) of the MONUSCO. The flyer was also translated in French and Swahili.


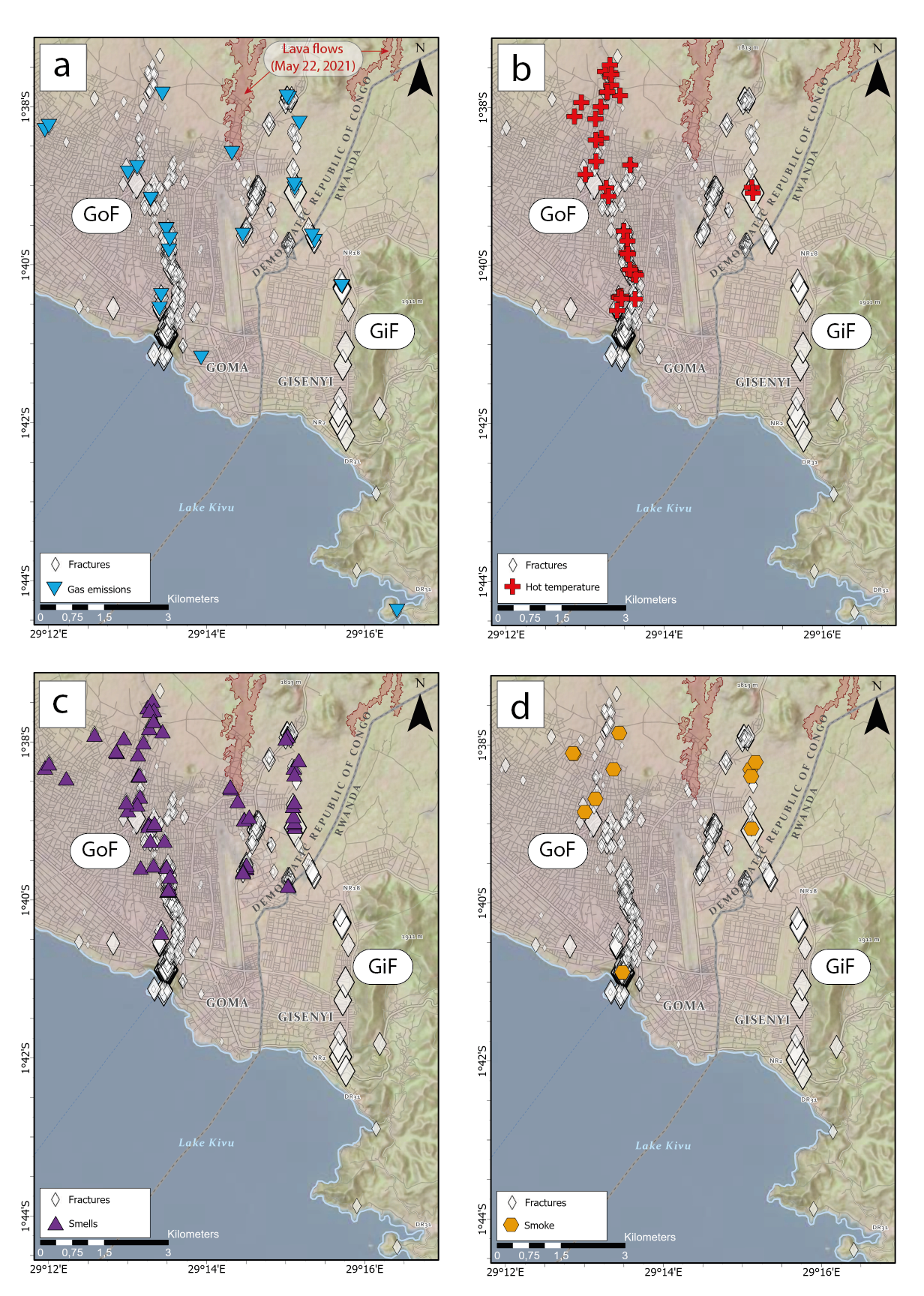


Fig. S2. Maps produced from the results of the participatory system that refer the location of the various anomalies (smells, hot temperature, gas emissions, smoke) identified by local populations. Credits Attribution: Esri, NASA, NGA, USGS; RMLUA, OpenStreetMap, HERE, Garmin, METI/NASA.


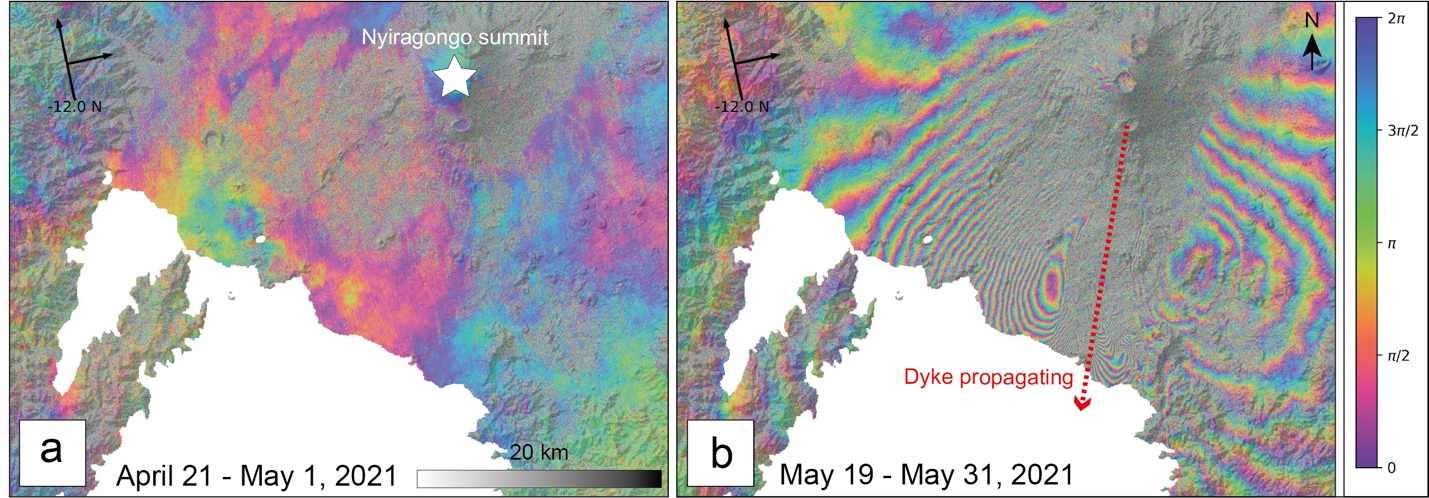


**Fig. S3.** Interferograms showing (a) the absence of deformation and (b) the large-scale deformation pattern that displays two lobes with opposite displacements directions, typically interpretated as the result of a dyke intrusion along the symmetry axis^20^. The propagation path is therefore interpretated to be nearly North-South, similar to the network of fractures reported in this study.

Data S1. List of the fractures and anomalies referenced by the population through the participatory system.

Data S2. Gas and temperature measurements performed on some fractures previously identified by the local inhabitants.
